# Making Cities Smarter—Optimization Problems for the IoT Enabled Smart City Development: A Mapping of Applications, Objectives, Constraints

**DOI:** 10.3390/s22124380

**Published:** 2022-06-09

**Authors:** Abbas Shah Syed, Daniel Sierra-Sosa, Anup Kumar, Adel Elmaghraby

**Affiliations:** 1 Department of Computer Science and Engineering, University of Louisville, Louisville, KY 40208, USA; anup.kumar@louisville.edu (A.K.); adel.elmaghraby@louisville.edu (A.E.); 2 Department of Computer Science and Information Technology, Hood College, Frederick, MD 21701, USA; sierra-sosa@hood.edu

**Keywords:** smart cities, Internet of Things (IoT), Artificial Intelligence, optimization, genetic agorithm, particle swarm optimization, heuristics

## Abstract

One of the prime aims of smart cities has been to optimally manage the available resources and systems that are used in the city. With an increase in urban population that is set to grow even faster in the future, smart city development has been the main goal for governments worldwide. In this regard, while the useage of Artificial Intelligence (AI) techniques covering the areas of Machine and Deep Learning have garnered much attention for Smart Cities, less attention has focused towards the use of combinatorial optimization schemes. To help with this, the current review presents a coverage of optimization methods and applications from a smart city perspective enabled by the Internet of Things (IoT). A mapping is provided for the most encountered applications of computational optimization within IoT smart cities for five popular optimization methods, ant colony optimization, genetic algorithm, particle swarm optimization, artificial bee colony optimization and differential evolution. For each application identified, the algorithms used, objectives considered, the nature of the formulation and constraints taken in to account have been specified and discussed. Lastly, the data setup used by each covered work is also mentioned and directions for future work have been identified. This review will help researchers by providing them a consolidated starting point for research in the domain of smart city application optimization.

## 1. Introduction

The increasing population and urbanization in the world has led to increased stress on cities around the world. With an estimated 70% of the worlds population living in cities by 2050 [1], governments and administrations are developing methods to cater to this increasing rise in their city’s dwellers. Moreover, the ever increasing effects of climate change and global warming have made necessary that these developments to the city’s capacity also be sustainable and environmentally friendly, following the United Nations Sustainable Development Goals for 2030 [2]. Considering these requirements, Smart Cities have become a topic of great interest by governments and the private sector worldwide. Smart Cities make use of technology to improve the living experience of the city’s inhabitants by contributing to important aspects of a city’s functioning. There are various domains within smart cities, these include Smart Agriculture, Smart City Services, Smart Grid, Smart Health, Smart Homes, Smart Industry, Smart Infrastructure and Smart Transportation. With improvement of Artificial intelligence (AI) capabilities in the last decade, there have been different applications of machine and deep learning algorithms within each of these domains [3] providing for better decision making and improvement of services. Apart from various supervised and unsupervised learning applications, several tasks within different components of smart cities can be formulated into optimization problems and/or require heuristics to be incorporated in some form. However, coverage of such efforts for IoT bases Smart Cities has received less attention compared to applications utilizing algorithms within the machine and deep learning domain.

In this regard, this paper presents a coverage of combinatorial optimization in Internet of Things (IoT) based smart cities by deliberating on the most popular applications of optimization algorithms and providing an insight to how those problems are formulated and worked upon. Since optimization has been a topic of interest to researchers in general, there have several surveys which pertain to individual aspects of smart cities such as electricity distribution networks [4], emergency facility location [5] and optimization in the industry [6]. Vukobratović et al. in [4] discuss the various optimization schemes used in distribution network management and scheduling, Wang et al. [5] provide an overview of the emergency facility location problem. They deliberate on the mathematical formulations and the extension of those problems. In [6], the authors provide a review of optimization algorithm development for industry 4.0, they provide a discussion of the problems present in the industry and also discuss mathematical formulations. Our work is different from the work in [4,5,6] in that we provide a mapped overview of the optimization landscape in the smart city domain while considering the IoT. Through this mapping, the common optimization problems across the various components of the IoT enabled smart city have been identified to assist researchers working on optimization schemes in the field. For each problem discussed, the objective function used, the nature of the objective as well as the constraints considered have also been elaborated on. As has been observed by [7], combinatorial optimization problems in the real-world are known to be difficult to formulate and generally are hard to solve. Moreover, choosing the right algorithm is also a tedious task as each comes with a different set of characterizations. This is very applicable to the IoT bases smart city where applications in each component caters to a different environment and aspect of the city’s operation and therefore requires intricate design of the problem.

In order to organize the review, this paper takes guidance from the work of [7]. They note that the most popular algorithms for use in combinatorial optimization problems are the Ant Colony Optimization (ACO), Genetic Algorithm (GA), Particle Swarm Optimization (PSO), Differential Evolution (DE) and Artificial Bee Colony (ABC). Moreover, following from the discussion in [7], this paper considers six factors for each application identified. These are:

1. The type of application: This refers to the problem being optimized within the smart city domain.
2. Data Setup: For each application, we mention the data setup used. While doing so, we aim to capture the various ways in which researchers have sourced data for their proposed method.
3. Single-Parallel problems: Another thing to note in smart city optimization problems is whether a problem has been considered as a single problem or multiple sub-problems/parallel.
4. Objective direction, function and number of objectives: Maximization or minimization, lowest fitness function value desirable or higher fitness function value is desirable. Since we list the objectives, we also covere the number of objectives inherently. Single objective, where a single fitness is optimized for its best value or multi-objective where multiple objective functions need to be considered at the same time. The latter is a complex process as some objectives may have conflicts and thus requires the need to perform trade-offs with what’s acceptable.
5. Constraints: Constraints are a set of restrictions or prerequisites that may sometimes be necessary to determine if a solution is considered valid or not. Soft constraints are desirable but not necessary whereas hard constraints are mandatory to be met. Constraints are put on the fitness function according to application being considered. Covering this aspect is particularly important as constraints are determined by the application being worked on.

The main contributions of the paper are as follows:

1. The paper provides a mapping of the optimization scenario for IoT based smart city applications for all smart city constituent domains thereby providing an overall view of the state of IoT enabled optimization applications for smart cities.
2. For all of the applications discussed, the objective/s used in the problem formulation are identified in terms of what function was used, the number of objectives, whether it was solved in a parallel or singular manner as well as the constraints considered have also been highlighted.
3. The detailed information provided herein for the covered work helps highlight the diversity of the formulations used in different smart city applications. As is observed, similar applications in smart cities require significantly different formualtions in terms of the constraints imposed as well as the objective function utilized. Using the provided insights, researchers and other stakeholders working in the field of smart city optimization will have a firm starting point to develop new applications.
4. It provides recommendations and directions for future work in the domain of using optimization algorithms for the IoT based Smart Cities.

The survey is organized as follows, Section 2 presents an introduction to the five considered combinatorial optimization algorithms, Section 3 presents the applications found for these algorithms for each component of smart cities and provides a discussion of them based on the factors discussed previously. Recommendations based on the compiled coverage are given in Section 4 with directions for future work in Section 5 to help guide researchers in this field while Section 6 concludes the paper.

## 2. Algorithms

Metaheuristic algorithms are widely used to solve combinatorial optimziation problems in the real-world [7]. The aim of these algorithms is to determine the minima or maxima of an objective function which translates an optimization objective in to one or more mathematical equations. Five algorithms have been considered in this review, these are the Ant Colony Algorithm, Genetic Algorithm, Particle Swarm Optimization Algorithm, Differential Evolution and Artificial Bee Colony. As mentioned earlier, these have been chosen as these were the most commonly used optimzation algorithms identified by [7]. In this section, we provide a brief intuitive description for each of them.

### 2.1. Ant Colony Optimization

Ant colony optimization is derived from the behavior of ants searching for food [8]. It was observed that during this process, each ant deposits a chemical called pheromone on the path which it takes towards the food. The more the ants take a given path, the more the pheromone is deposited on it as it is deposited by each ant going over it. Subsequent ants that want to go towards the food use the amount of pheromone on a given path or sub-path to decide which path to take so as to determine the optimal route to the food. In the artificial ant colony optimization algorithm, the points on the path that form the sub-paths are encoded on a graph where each ant can only visit a given vertex (travel on the same sub-path) once. Each iteration starts with a number of artificial ants, an ant would choose the next vertex to visit based on the level of pheromone on the possible sub-paths available to it as well as whether that path has been used before. At the end of each iteration, the pheromone levels are updated so as to prioritize the use of the most used path for ants in the next iteration.

### 2.2. Genetic Algorithm

A genetic algorithm [9] is based on evolutionary science. The idea behind the genetic algorithm is that starting from a given population set of organisms, subsequent reproduction will result in fitter organisms being produced given that the organisms chosen for reproduction have some level of fitness. To solve an optimization problem, a genetic algorithm begins with a given population size of strings also called a chromosome. Each ‘chromosome’ consists of a ‘gene’ which may represent points in the population. The sequence in which the genes are present would represent a solution to the problem. For e.g., for a route scheduling problem, this may be the coordinates of the stops. The ‘goodness’ of a chromosome is determined by a fitness function that quantifies how appropriate a given chromosome is as a solution for the problem. Choosing the right fitness function is an important consideration in genetic algorithms as it needs to gauge the kind of optimization that is to be performed. The optimization process starts with an initial population of a given size. Once a fitness function has been defined, in each iteration, two or more chromosomes (parents) are taken at random from the population and one or more offsprings are generated. The random selection takes the selected parents fitness function value in to account, however, its necessary that not all parents chosen are the fittest of the population as otherwise, diversity will be compromised, and the algorithm may get stuck in a local minimum. The method by which these offspring are generated is called the Crossover function and the number of parents mated to form offsprings from them depends on the crossover rate. The Crossover function defines the way the genes within the chromosomes are exchanged. Usually, the crossover rate has a high value. Moreover, depending on some mutation rate, a given fraction of all offsprings are mutated. Mutation depends on the mutation function used and involves members of the offspring being swapped in some manner. When one iteration of the offspring creation from the parents in the entire population is completed, the offsprings replace members of the original population (typically the weakest) and one generation of the GA is said to be completed. In order to converge to a sufficiently good result, GA’s need to run for many generations.

### 2.3. Particle Swarm Optimization

Particle Swarm Optimization is modeled on the social behavior of animals rather than the evolutionary biological behavior on which Genetic Algorithms are based. PSO [10] is particularly based on the behavior of a flock of birds searching for food. Each bird in the flock searches for food and can communicate with other birds in the flock as soon as it finds food or a good direction for it. Therefore, each bird has two food ‘directions’ that it needs to consider, first is on an individual level which is called the personal best and the second is on the flock level which is the global best. Using these two values the bird will proceed towards that path and will inform other birds in the flock too. The idea here is that after enough time and with all the individuals working together, the swarm will find the place with the highest concentration of food. In terms of using PSO for optimization tasks, individual birds are equivalent to a solution as in a GA and each is considered as a point or a particle, collectively they make up the swarm. This swarm population may be initialized randomly or based on some domain knowledge about the problem. The highest concentration of food represents the optimal solution for the whole swarm where as a good direction represents the local optimal solution for each case. The aim here, like birds in a flock is to determine the global optimal solution using information from each individual particle. Each particle has three aspects to it, its position, its velocity and a value of its current position’s ‘goodness’. This ‘goodness’ as in the GA is defined by a fitness function. Like birds, each particle has a personal best fitness value and is also ware of the global best fitness value. For any particle, the new direction of movement is decided by a combination of the personal best and the global best as well as the particle’s intention to maintain its current movement. Furthermore, several different topologies can be utilized, and a neighborhood can also be set for each particle to convey positions when limiting the global best parameter to the local best scheme rather than the whole swarm. The algorithm may be stopped till the best solution is reached or no more improvement is observed.

### 2.4. Differential Evolution

Differential evolution [11] is a stochastic evolutionary algorithm which is used for optimization problems for continuous functions. The DE algorithm does not expect gradient information and thus it doesn’t need to be differentiable. Like other evolutionary algorithms, a solution is searched for in the design space by maintaining a set of individual candidate solutions. In each iteration, the solutions with the best fitness are combined together and retained for the next iteration. The aim is to improve upon the fitness value and this process is repeated until a pre-decided condition for termination of this process is satisfied. Differential Evolution is very similar to Genetic Algorithms in that both are evolutionary in nature, however, the difference is that the DE uses real numbers in the chromosome and also that the mutation operation consists of the difference between two or more chromosomes of the population with the possible addition of some type of noise to create a new individual. Like GA, DE also suffer from getting stuck in local minima. The DE algorithm also has three control parameters, the population size, the mutation factor and the crossover probability.

### 2.5. Artificial Bee Colony

Article Bee Colony [12] is a swarm intelligence algorithm based on the behavior of bees. Within bees, there are different social classes who work together to complete tasks such as harvesting pollen and nesting through the use of smell, ’swing dance’ and other methods. Bee colonies consist of three types of bees, queen, male and the worker bees which work on activities such as searching for food, gathering and storage of honey. After gathering honey, the worker bee comes to the nest and informs other bees about the location of the honey source site through a dance. The ABC algorithm simulates the behavior of bees by considering three elements, the food source, employed bees and unemployed bees. The food source is represented as revenue considering its distance and quality, the higher the revenue, the better is the value. In computational optimization terms, the food source is a potential solution to the objective formulation of the considered problem and the quality or value of the food source represents the fitness value of that particular solution. At the start, all bees are used as scouts, searching for food sources. When a food source is found, for a high value food source, bees who find those food sources become hire bees and collect the food source. If the food source is of intermediate value, the bees become follow bees and if the food source value becomes low, the bees become scout bees to look for better food sources. Hire bees, the bees with a food source location having a high value, lead the follow bees to develop solutions in their neighborhood in order to come up with more solutions. A subset of the highest-ranking solutions are then considered before this process is repeated again until the end conditions are met.

## 3. Optimization Application in Smart Cities

Several tasks in smart city operations require the use of metheuristics to be solved, the aim being to optimize the resources present in the city. This section presents the different uses of optimization techniques for IoT enabled Smart Cities. This task is performed for all eight components, Smart Agriculture, Smart City Services, Smart Grid, Smart Health, Smart Homes, Smart Homes, Smart Industry, Smart Infrastructure and Smart Transportation.

### 3.1. Smart Agriculture

**Figure 1:**
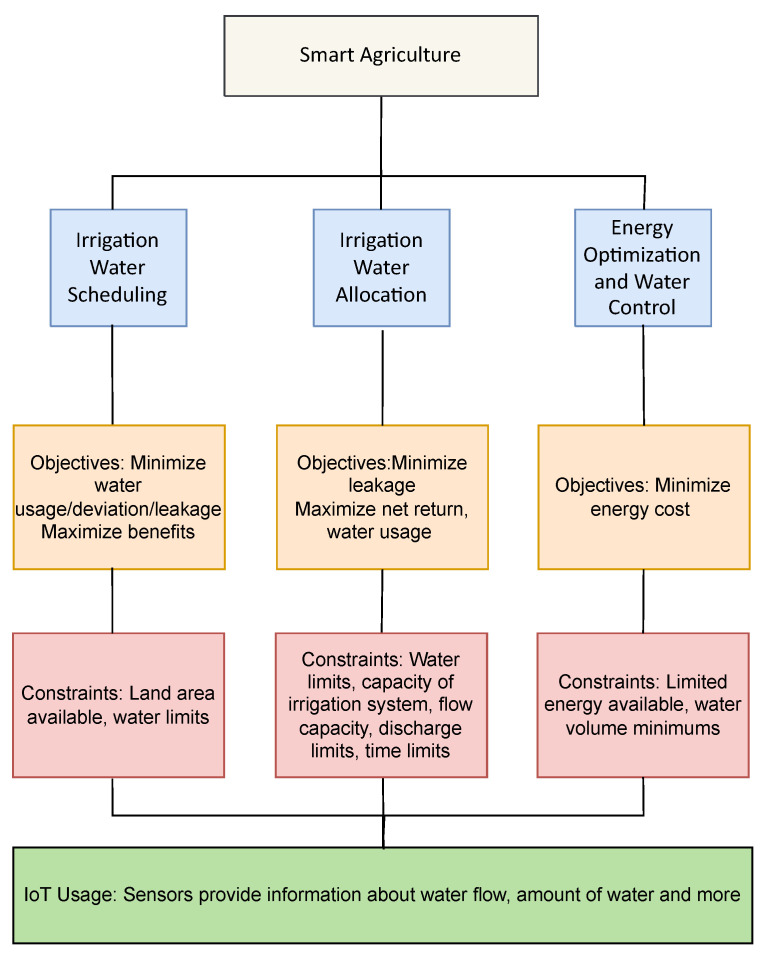
Optimization applications in Smart Agriculture.

Smart Agriculture involves the use of digital technologies such as sensors and intelligent systems to improve agricultural productivity. The sustenance of agriculture depends on water, and it is central to the agricultural production of food items around the world. However, water is becoming an ever-scarce resource due to the increasing demand caused by human population growth, increased economic activity by industries and also due to climate change. It therefore is necessary to utilize this precious resource wisely so as make use of it in the best manner possible. One approach towards ensuring that water and land is used appropriately is by introducing irrigation management schemes such as irrigation scheduling and water allocation in the farming process. A summary of the optimization problems, objectives, constraints in smart agriculture and the use of IoT is illustrated in Figure 1.

Measurements in water irrigation systems are typically performed by sensors placed at different points in the canals and rivers to determine water flow, volume and speed. This information regarding water movement can be combined with economic information such as yeild, crop profit to optimization water distribution. Irrigation management through scheduling has been performed by the authors of [13,14,15,16] to maximize net return on crop profits, water use efficiency and to minimize leakage losses. In [17] Fuqiang et al. aim to optimize water delivery through canals while also performing scheduling. They do this using a genetic algorithm and work with two objectives, minimizing the difference between the time of water delivery and water demand and the fluctuation in water discharge of the main canal. Their model was applied to a district in China.

The authors in [18,19] work on optimal allocation of water. Of these, Ikudayisi et al. [18] use the differential evolution algorithm to minimize the water allocated to farms in South Africa while maximizing the benefits in terms of job creation, ensuring food security and the yield of crops. Wu et al. [19] approach this as problem of reducing deviation between different channels and minimizing water seepage to ensure a more consistent supply to all water channels. An approach presented by Ocampo et al. [20] tackles this problem not as a task of allocation directly but considers the scenario of providing sufficient water to a smart farm while controlling two water pumps. The objective function is formulated to minimize the energy cost of the water pumps while maintaining sufficient supply of water to the plants on the farms. Constraints considered for such applications include the limited quantities of water being worked with, the time for which the water was available and the area of land which was to be considered. Another allocation based scheme is presented by the authors of Zhuo et al. [21] who use a genetic algorithm for an irrigation system based on a reservoir and two pumping stations. Their aim is to ensure that there is no water shortage. The objective function used by them is the minimization of the annual sum of squared water shortage. Other work in [22] also minimizes use of groundwater and increase benefit to the regional water supply through reduction of water deficits in the Dujiangyan of China.

A precision agriculture scheme is presented by Roy et al. [23] who combine an IoT system consisting of sensors on plants using a GA based rainfall predictor. Combining predicted rainfall information along with the sensed moisture level in the crops, they control plant watering. Lin et al. [24] propose a framework for the management of fertigation and irrigation in precision agriculture. They perform short term management and long-term management. Soil and crop growth data is gathered from IoT based sensor systems. Long term planning refers to the allocation of water and fertilizer resources to crops in terms of quantity so as to meet demands whereas short term refers to when how to use them. They use a genetic algorithm for long term planning considering the allocation of water and fertilizer for crops to maintain pH value and the electrical conductivity. This information has been presented in Table 1 while a summary of the identified data sources used by each considered work has been provided in Table 2.

### 3.2. Smart City Services

**Figure 2:**
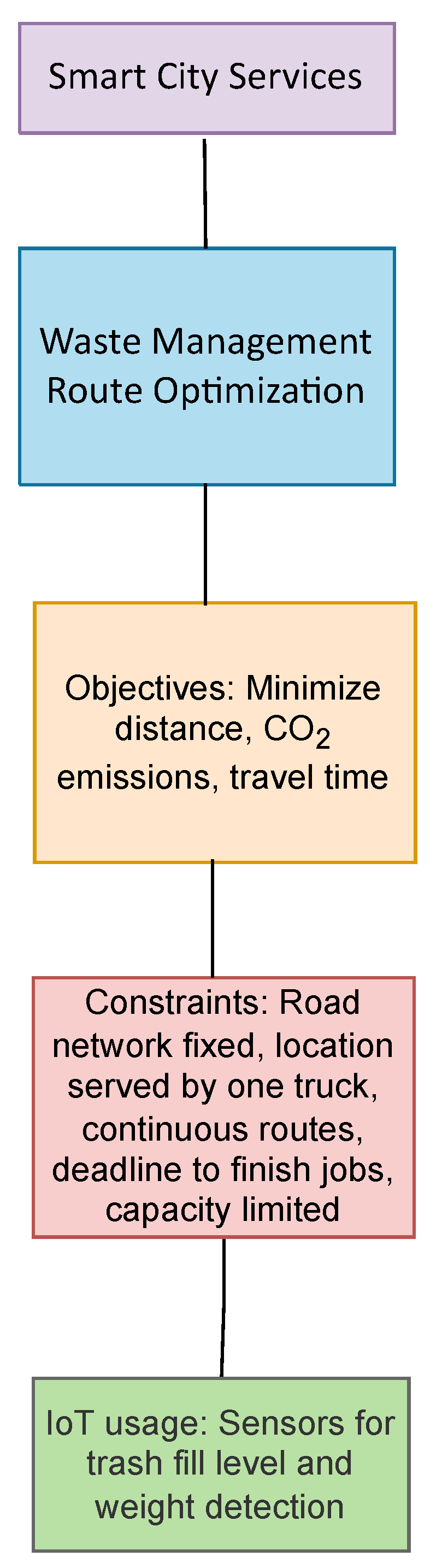
Optimization applications in Smart City Services.

According to the world bank, the amount of annual solid waste generated is set to be 3.40 billion tons [26] in 2050. Managing this waste and its collection in an efficient manner is imperative for health and climate reaons. The most common application towards smart city services optimization is waste management as illustrated in Figure 2 which summarizes the objectives, constraints and the use of IoT.

Smart waste collection systems include sensors attached to trash cans which can inform the municipal authority about the status of the garbage present in them. Once the trash cans are close to being full, it is the responsibility of the municipal corporation to perform garbage collection in an efficient manner. In this respect, data provided by the sensors on garbage cans can be used to determine an optimized route for garbage collection to construct the Vehicle Routing Problem (VRP) in the Smart City Services domain. As such, this problem has been performed keeping in view various goals. The minimization of the route distance taken by a garbage tuck has been performed by the authors in [27,28,29,30,31]. The aim in this case is to determine a route for garbage collection vehicles that minimizes the total distance traveled by the them. Zhang et al. [31] consider multi-vehicle allocation while considering the single objective of minimizing route distance. Wei et al. [32] use the Artificial Bee Colony algorithm to determine garbage collection routes resulting in the minimum emission of CO${}_{2}$. Another optimization objective in route optimization for waste management has been the minimization of the total travel time, such a target is described by the authors of [33,34,35,36] who aim to reduce travel time while considering emptying of waste bins. Another optimization consideration in route optimization for waste management is to reduce cost. This has been carried out by Tirkolaee et al. [37] who formulate a multi-objective function of travel cost and total useage cost of vehicles and determine the route with the minimum costs using the ACO. Constraints considered in such applications are related to a fixed road network which depends on the locality for which the optimization is being performed, the continuity in the determined route as well as fulfillment of capacity restrictions. The useage of optimization algorithms in smart city services is provided in Table 3 and the data sources used are provided in Table 4.

### 3.3. Smart Grid

**Figure 3:**
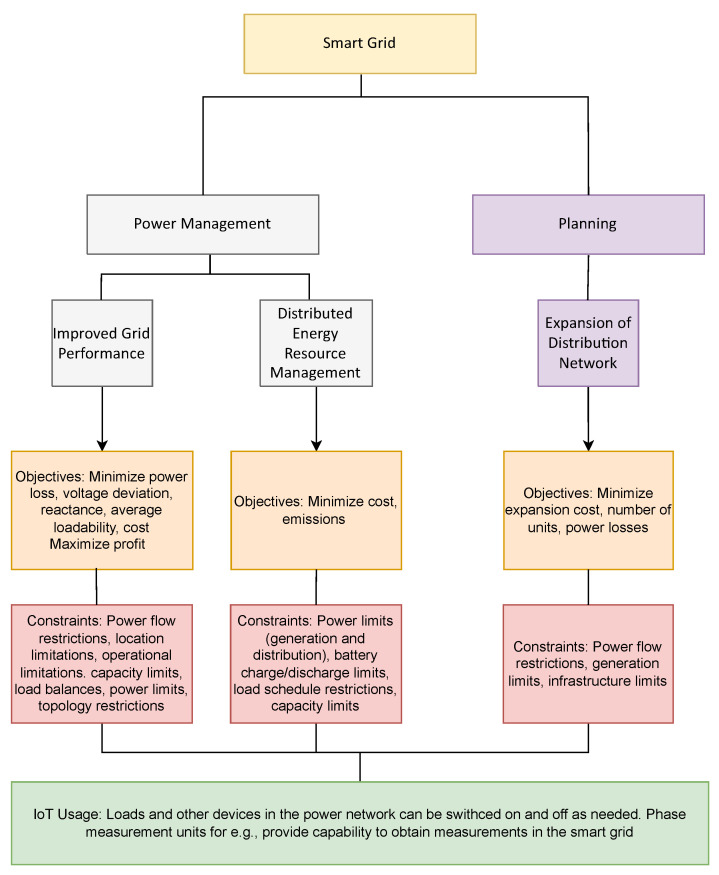
Optimization applications in Smart Grid.

The electricity grid has been a major beneficiary of smart city technologies. The increasing demand for energy by consumers along with the environmental impact that fossil fuel-based energy production has on the planet has forced utility companies to introduce renewable energy sources within the electricity distribution system and make their energy production and distribution systems more efficient through planning and design improvements. Optimization algorithms find applications within the smart grid (SG) domain in terms of power management and planning. A summary of the applications, objectives, constraints and IoT useage for optimization algorithms in Smart Grids has been illustrated in Figure 3.

An increasing population has led to an increasing demand for electricity around the world. This burdening of the electricity grid has led to measures for increasing the performance of the electricity distribution system by reducing loss, prevent overload and reduce cost. The authors in [40,41,42,43,44,45,46] work on the improvement of grid performance by minimizing cost and reducing power losses. Power loss minimization is specifically targeted by [40,42,44]. Of these, Ettappan et al. [40] aim for the reduction of power losses, voltage deviation and increasing voltage stability. Atteya et al. [44] also address this problem by considering network redistribution to minimize losses in the grid whereas Sakr et al. [42] focuses on minimizing transfer losses in the smart grid to accomplish this task. Nguyen and Mohammadi [43] attempt the reduction of power losses and line congestion by determining the location of thyristor controlled series compensator devices (TCSC). The problem is formulated as a multi-objective problem aiming to minimize loadability of the lines, active power loss and the reactance of the transmission line. A cost reduction-based approach to improve grid performance is followed by Das et al. [41] who aim to reduce cost of maintaining electrical stability and also the cost of management of the distribution network. The do this by considering changing the location of energy storage systems within the grid. Kanwar et al. [45] take maximizing profits and minimization of power losses while considering sizing of a distributed energy resource generation system.

Distributed energy resource (DER) management is another area where optimization algorithms are used in Smart Grids. The management of distributed energy sources within smart grids is dependent on the interconnectivity provided by IoT in the smart grid system. Smart meters within the smart grid provide real-time information relating to power consumption which can be used for controlling DER electricity. Moreover, IoT devices allow for switching loads and generation sources as required. This assists in creating a virtual power plant (VPP) to aggregate all energy sources in a DER scenario. With global warming and a changing climate, utilities around the world are increasingly incorporating various renewable energy sources within their grid which often times are an economically convenient option as well. However, many of these sources such as wind and solar (photo voltaic [PV]) do not offer a consistent supply of power throughout the day. In this regard, systems such as batteries as well as conventional generation plants need to be used together along with renewable energy sources. For utility companies, it is necessary to optimize power production so that the maximum amount of energy is utilized from these renewable sources so as to reduce cost to the user while also maintaining the quality of service. The authors in [47,48,49,50,51,52,53,54,55,56,57,58,59,60] provide a management scheme for DERs to minimize cost. In this regard, the authors in [47,48,50,51,52,55,58,59,60] all formulate the problem of distributed energy resource management as a single objective problem where the cost incurred is minimized. On the other hand, the authors in [49,53,56,57] formulate this as a multi-objective problem. Azaza and Wallin [57] not only target reduction of electricity production cost but also maximize reliability of the system while reducing the environmental impact of the distribution system. It is interesting to note that the improvement of system reliability is formulated as a minimization problem as well. Similarly, Das et al. [49] consider the reduction of both the total cost as well as the environmental cost of the system. Their considered scenario also consists of a PV, Wind Turbine and battery. The constraints considered were constraints regarding power flow, limitations on power and voltage values, power balance constraint and power generation constraints on the renewable energy sources. In [47,60], a DER management system is developed for a microgrid which consists of a controllable collection of energy storage and generation sources powered by IoT devices.

Planning in distribution networks has been considered by the work of [61,62]. Mahdavi et al. [61] work on expanding transmission lines utilizing the artificial bee colony algorithm to minimize cost of network expansion, power losses in load and generation. On the other hand, Maji and Acharjee [62] aim to determine the minimum number of Phase Measurement Units (PMUs) to make the distribution network observable. The constraints used were power flow and balance of power as well as limits on the number of transmission lines available. The internet of things also finds usefulness in terms of the use of Phase Measurement Units (PMU) that provide voltage and current measurement capabilities within smart grids to perform maintenance and monitoring operations. This has provided in Table 5 and the data setups used by the covered research work is presented in Table 6.

### 3.4. Smart Health

**Figure 4:**
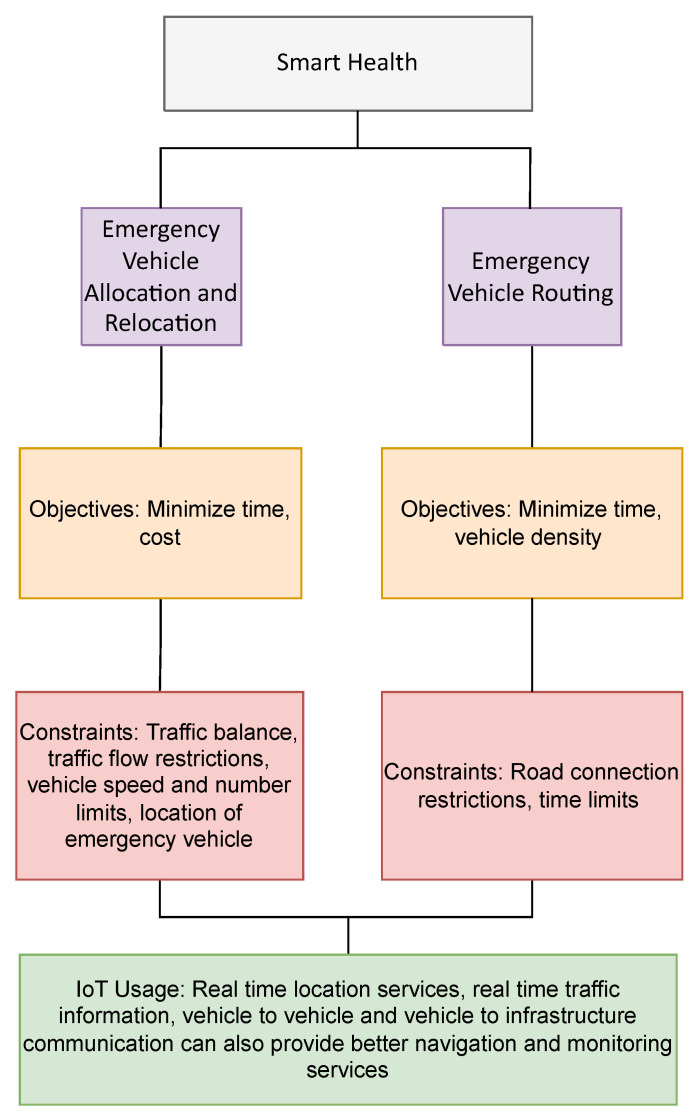
Optimization applications in Smart Health.

Smart health refers to the use of technology to provide better healthcare to patients. This can be in the form of developing tools for better diagnosis of diseases or the use of algorithms for better planning and healthcare delivery. The deployment of timely emergency vehicles to a person in need is imperative towards providing healthcare services to people. Two applications of optimization problems within Smart Health are emergency vehicle routing and their allocation and relocation as shown in Figure 4. It also summarizes the objectives uses, constraints considered and role of IoT.

Late arrival of ambulances and other emergency vehicles to people in need may result in irreversible damage to life and property. Studies have shown that delayed ambulance dispatch increases mortality [65], moreover, economically speaking, a one-minute delay in response time for cardiac patients found that the mortality increases by 1% and adds annual costs of USD 7 billion in healthcare expenditure [66]. Keeping this in mind ambulance deployment and location determination have been of considerable interest in the area of optimization for smart health. These two problems are specific cases of the Vehicle Routing Problem [67] and Maximum coverage problem [68] sometimes called the Ambulance Routing Problem [69] and Ambulance Location Problem [70]. The authors in [71] work on the optimal allocation determination based on fixed sites and a finite number of ambulances while minimizing lateness of ambulance arrival using the Ant Colony Optimization. Later on, in their work in [72], they do a comparison with using GAs and find that GAs provide the better performance. Kochetov and Shamray [73] attempt localization of ambulance fleet at base stations with the aim to minimize the average waiting time for arrival of ambulances. An interesting approach to this problem is presented in Yan et al. [74] who work on this problem from a scheduling perspective where they control scheduling of emergency vehicles to reduce the total cost in terms of money and time using a Genetic Algorithm. Another approach for sequencing vehicles to ensure emergency vehicles reach their destination in time is presented by Lu et al. [75] who aim to prioritize emergency vehicle thoroughfare on traffic intersections. They do this by minimizing the entrance time of the vehicle by manipulating vehicle order. Constraints used for these problems include constraints on the speed of the ambulances, the flow of vehicles on the road, specific road connections present as well as time constraints. The internet of things serves a pivotal role in enabling the allocation and routing of emergency vehicles. The connectivity provided by IoT through vehicle-to-vehicle communication as well as vehicle to infrastructure communication facilitates providing a real-time indication of the vehicle’s location as well as the condition of traffic in a given area. This information can then be used to determine an optimal route for emergency vehicles as well as for their optimal deployment to serve people in need. Information about optimization methods for smart health has been presented in Table 7 and the data setups used in these approaches in Table 8.

### 3.5. Smart Homes

**Figure 5:**
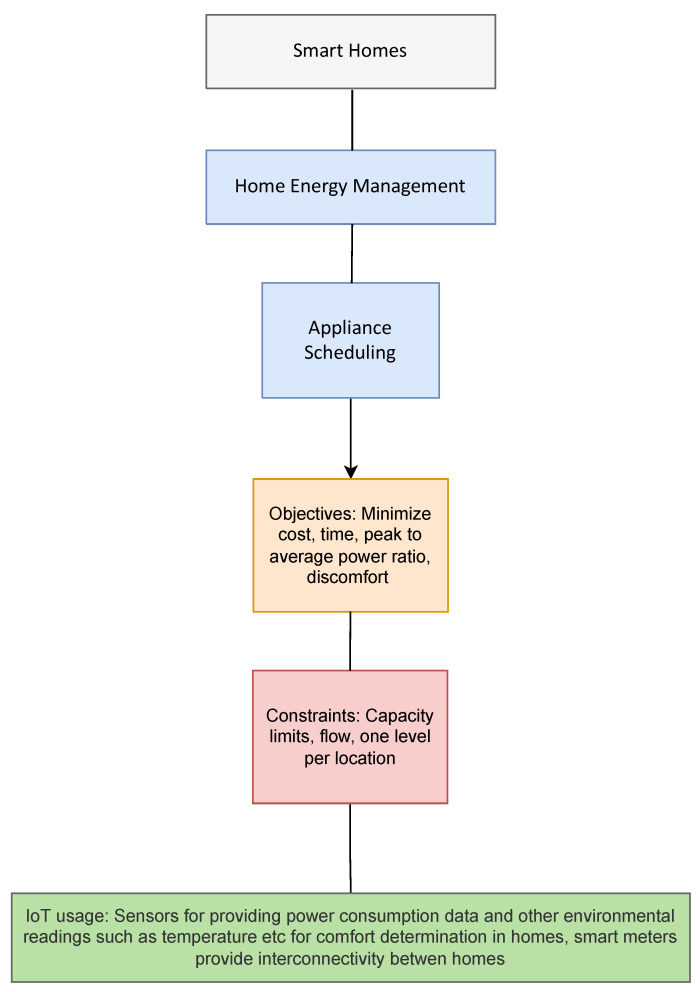
Optimization applications in Smart Homes.

Home energy management has been the prime application of optimization in smart homes, a summary of the objectives, constraints and the use of IoT has been shown in Figure 5.

Home energy management refers to the development of demand side management schemes that aim to reduce the electricity cost billed to a customer or maintain comfort for the user. One way this is performed is by appropriate appliance scheduling. The idea here is to schedule the usage of appliances in such a way that the most power-hungry devices are turned on during off peak hours when electricity costs might be lower. The combination of the Smart Grid and Smart Homes facilitates the development of optimization schemes that not only benefit the customer (in terms of reduced electricity costs and maintaining comfort) but also be useful for the utilities in ensuring that load profiles (though minimizing the peak to average ratio) are more consistent thereby allowing better planning of the power generation mix used by them. The authors of [77] perform appliance scheduling for the purpose of minimizing electricity cost and the waiting time for appliance usage. Interestingly, they incorporate comfort maintenance by adding it as a constraint. A similar approach has been followed by Bui et al. [78] and Makhadmeh et al. [79] who aim to minimize the cost of electricity usage with a constraint for maintenance of comfort. Makhadmeh et al. [79] also include the reduction of waiting time rate for appliances by the user and the reduction of the peak to average ratio of the power consumed as constraints. The authors in [80,81,82,83] perform appliance scheduling while considering electricity cost and peak to average ratio which need to be minimized. All of the authors present a multi-objective function for this purpose combining the objectives of minimizing the cost and the peak to average power ratio. Azimi et al. [84] combine the problem of reducing cost and power together as a single objective by considering the minimization of the ratio of operating cost and load factor in a battery supported system. The works of [85,86,87,88,89] also consider user comfort as part of the objective. In [85], Essiet and Wang form a multi-objective minimization problem of electricity cost, peak to average ratio for power and discomfort of users in a smart home supported by a renewable energy system consisting of a battery and PV system. In Chanra et al. [90], the authors aim to reduce electricity cost by appliance scheduling in such a manner so as to make as much use of onsite energy units as possible so as to reduce usage of utility provided electricity. The energy units they consider are a diesel generator, renewables and battery. Another approach that aims to reduce cost of consumed electricity is presented by Faia et al. [91] who formulate it as a problem of minimizing the energy bill and the cost associated with curtailment of power in a system with a battery and a photovoltaic system. The work in [88,92,93,94] also perform appliance scheduling to reduce cost of electricity. Appliance scheduling for smart homes has also been performed by Fatima et al. [81] and Abid et al. [80] considering a microgrid for homes where instead of optimizing data from single homes, the authors used data from connected smart meters to determine an optimized control scheme for appliances across the grid. The constraints used for optimization in smart homes are on the comfort needing to be maintained, constraints on the powerflow, time of operation, the maximum power that is present or used and which appliances are switchable appliances. Appliance scheduling is based on smart meters as well as individual control and monitoring of appliances using IoT systems. IoT devices enable the microgrid which is used to gather data as well as control the switching on and off of sources from the houses electricty supply. The information gathered from these IoT units can be processed to optimize energy consumption patterns to reduce cost to the customer as well as increase comfort. The use of the considered optimization schemes for smart homes has been presented in Table 9 with the data setups presented in Table 10.

### 3.6. Smart Industry

**Figure 6:**
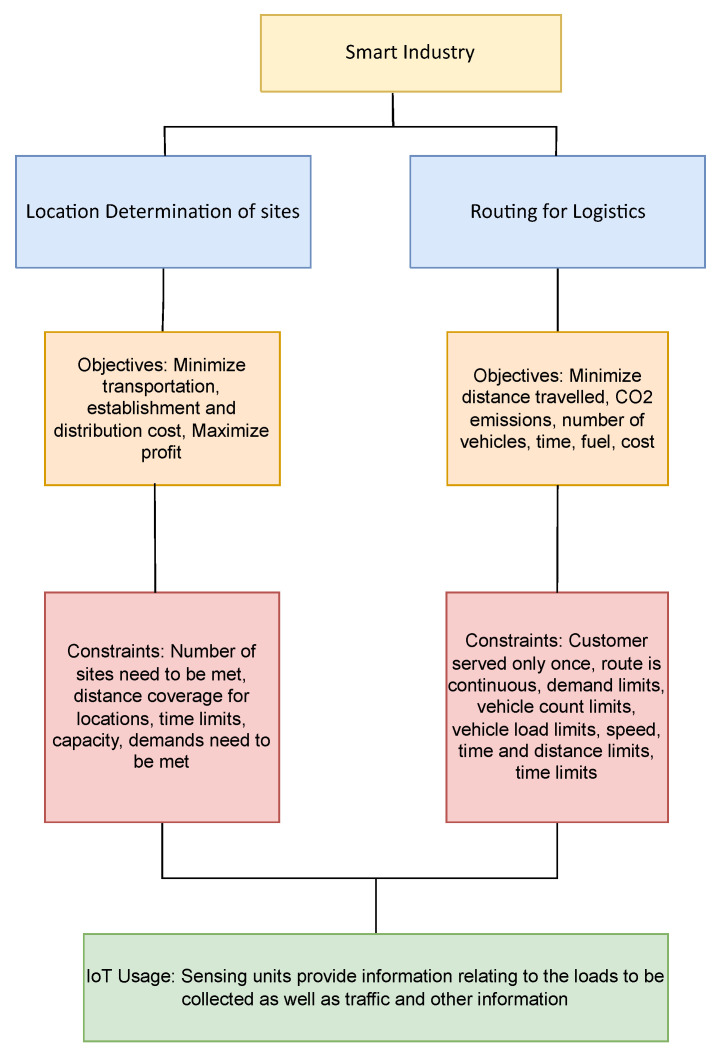
Optimization applications in Smart Industry.

One of the biggest enablers of the Industry 4.0 concept has been the use of AI techniques to improve the efficiency of the manufacturing and production process. This has led to the development of cyber physical systems aiming to assist in activity recognition [97], machine health prediction [98] and production management in terms of bottleneck prediction [99]. Apart from conventional AI applications of anomaly detection, classification and regression, computational optimization also finds numerous applications as it fits well with the objective of efficient and streamlined manufacturing. The major applications for the use of computational optimization have been in the area of routing and location for logistics and are variations of the vehicle routing problem and are typically represented as Multidepot Vehicle Routing Problem (MVRP), Vehicle Routing Problem Pick-up and Delivery with Time Windows (VRPPDTW) or Large-scale Dynamic Vehicle Routing Problem (LSDVRP). Figure 6 summarizes the objectives utilized, constraints and the role of IoT in optimization for Smart Industry.

The authors in [100,101] use the ABC and the GA respectively to determine the best location of service sites for logistic operations. Both these approaches use multi-objective formulations aiming to reduce cost of operations, transportation as well as the establishment of the centers. The authors in Su et al. [102] use ACO, Alinaghia et al. [103] PSO and Utama et al. [104] use ABC to address the problem of determining the best route for logistics operations. The routing and coverage problem for logistics involves determining the best route for either a single or multiple vehicles at a depot which have to visit every customer. The works of [102,103,104] focus on reducing the cost incurred in the routing for vehicles in logistics as a single objective formulation. On the other hand, the authors of [105,106,107] all work on the minimization of distance as their objective in determining the optimal route for delivery vehicles trying to serve multiple locations. Mounia and Bachir [106] address routing in logistics as a multi-objective problem where they not only aim to minimize the distance traveled by the vehicles but also aim to reduce CO${}_{2}$ emissions and the number of vehicles used. A time based optimization approach is presented by the authors of [108,109] also factoring in reduction of fuel consumption in their objective function formulation. Constraints used for the routing and location determination problem are related to time, capacity constraints for the vehicles, each customer being served only once, constraints related to the route. The determination of the location and the route for vehicles is dependent on real time information concerning the traffic in the area as well the loads to be collected from each site in addition to other information which can be provided by IoT units. The usage of optimization algorithms for smart industry has been presented in Table 11 with data setups presented in Table 12.

### 3.7. Smart Infrastructure

**Figure 7:**
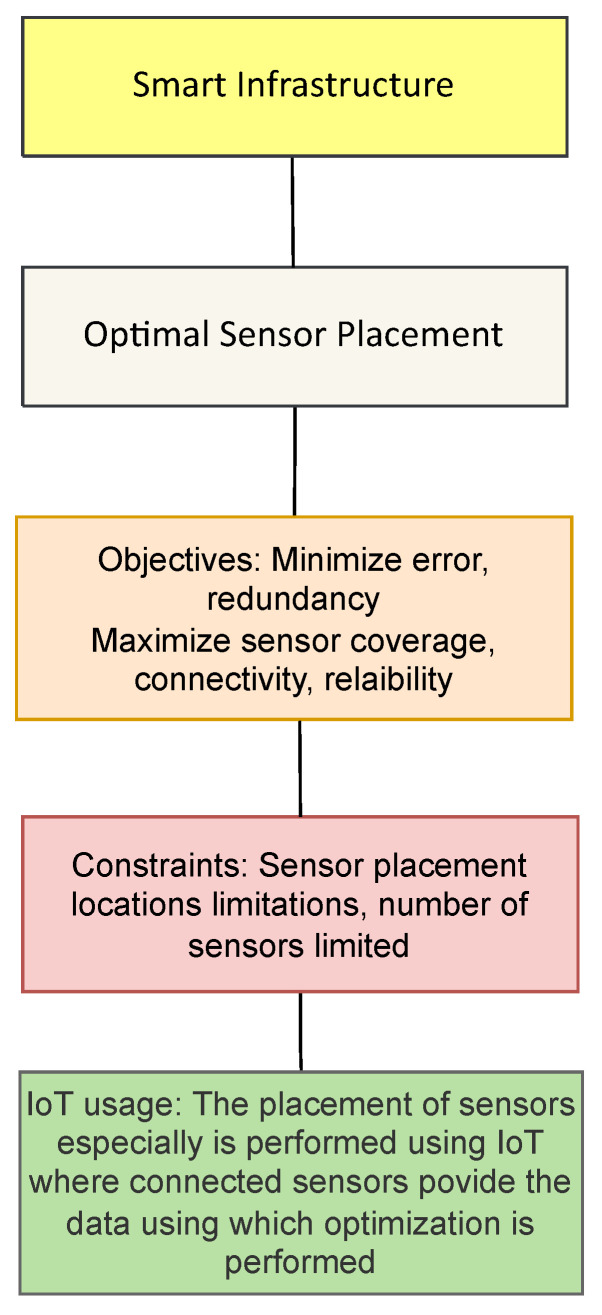
Optimization applications in Smart Infrastructure.

Within the infrastructure domain, the most common optimization problem is the area of health monitoring of structures. Structural Health Monitoring (SHM) is a necessary application within the smart infrastructure domain as it makes for safe usage of different structures of public use. These structures can be buildings as well as transport structures such as bridges, tunnels. Structural health monitoring typically involves the use of sensors attached to a structure at several points that can gauge some type of physical variable (vibration, strain, acceleration, temperature, tilt etc) from the structure. Data gathered from these connected sensors is then used to determine if any structural damage has taken place or not. Within the domain of SHM, optimization algorithms find application towards the Optimal Sensor Placement Problem (OSP) as illustrated in Figure 7. Figure 7 summarizes the objectives used, constraint and the use of IoT.

For the optimal sensor placement problem (OSP), the aim is to determine the best number and placement of sensors over a structure so as to reduce the number of sensors used as well as improve the measurement process, both these aims result in increased reliability of the SHM system as well as potentially lower the cost of the system too. The authors in [111,112,113,114,115] work on the placement of sensors for structural health monitoring focusing on improving the effectiveness of the deployed system. In this regard, refs. [111,114] use the genetic algorithm to solve a multi-objective problem aiming to minimize the measurement error and cost. Yang et al. in [113] formulate OSP as single objective minimization where they aim to reduce the ratio of sensor placement performance to the redundancy of information resulting from each tested placement. Another approach that works on the error is presented by [112] who use the Particle Swarm Optimization to maximize the reconstruction accuracy and robust transfer relationship between the deformation and surface strain with different sensor placements. It must be noted that the objective function is formulated as minimization of negated accuracy and negated robustness measurement. Optimized structural health monitoring for aircraft monitoring has been targeted in [116]. In their setup consisting of vibration sensors, the authors optimize sensor placement by minimizing the cross correlation of the vibration waves in the sensing network. The most common constraint for sensor placement is restrictions on the places where sensors can be placed. This information has been provided in Table 13 and the data setups are presented in Table 14.

### 3.8. Smart Transportation

**Figure 8:**
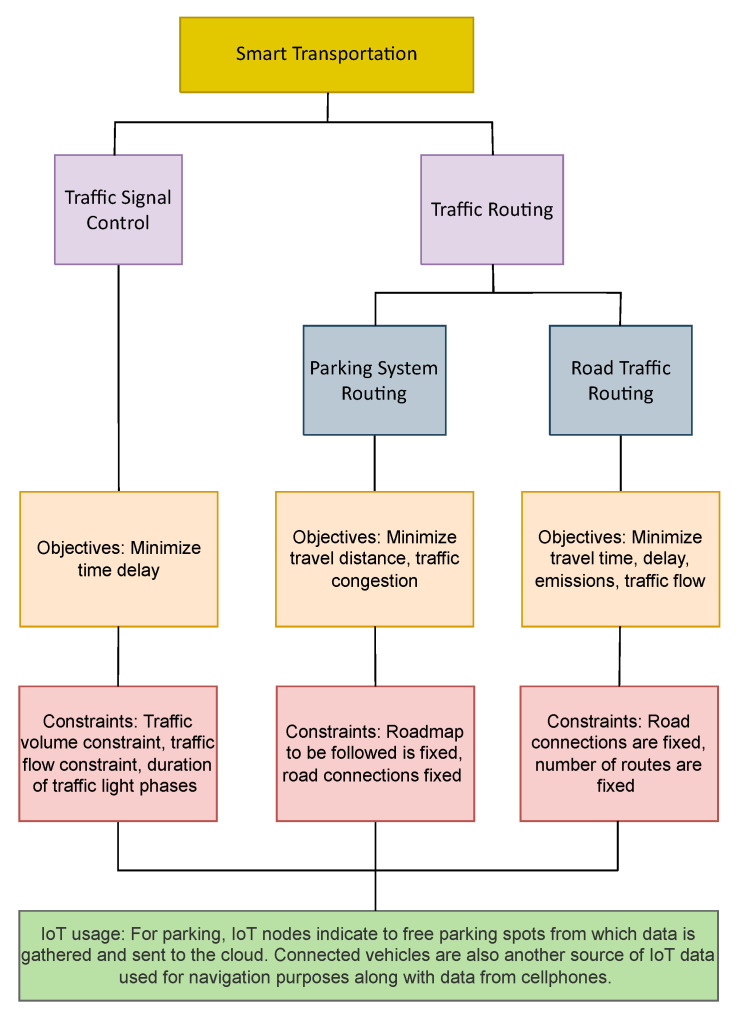
Optimization applications in Smart Transportation.

One of the most popular optimization applications within smart cities are within the smart transport domain. These include parking system routing, traffic signal control and scheduling. A summary of the applications, their objectives, constraints and the role of IoT is illustrated in Figure 8.

Smart transport systems consist of sensors along roads and traffic intersections to measure relevant parameters while also providing communication services between vehicles and infrastructure. This allows for measurement of the current state of roads in terms of traffic congestion and usage thereby allowing for the use of optimization techniques to improve trip experiences for users and make the transportation system more efficient. The authors in [119,120,121,122,123] work on the minimization of time (wait and travel) in traffic signal control. The aim of such systems is to reduce traffic build up on signal intersections. Of these, the work in [119,120,121] use the artificial bee colony and the genetic algorithm respectively for a single objective function of minimizing delay time. An interesting approach for this problem is presented by Li et al. [123] who use a multi objective formulation targeting the minimization of the average travel time both overall and individually for all vehiclesl. Another multi-objective approach in traffic signal control is presented by Chen and Yuan [124] who form a mixed problem of minimizing vehicle emissions and travel time together. Korkmaz [125] work on the estimation of delays in traffic signals using a genetic algorithm, they use it to minimize the difference between the estimated and simulated values. Tang et al. [122] carry out distributed optimization in a fog and cloud hierarchy. First, fog nodes optimize phase timings within a single cycle and if the number of vehicles exceeds a threshold, the results are sent to the central controller to further optimize over different cycles so that a traffic jam is avoided or alleviated. Zhang et al. [126] attempt traffic signal optimization using multi objective optimization functions of reducing time delay and increasing traffic capacity. Constraints used for traffic signal control are timing constraints on the phase durations, flow rate of vehicles and on the travel time.

Traffic routing is also another important aspect in smart transportation. This typically involves the determination of the best route to the destination keeping in view various criteria such as reduction of distance, time, cost etc. The problem of traffic routing is addressed by the works of [127,128,129,130,131,132,133,134,135,136,137]. The authors in [129,130] use the ant colony optimization and genetic algorithm to minimize the travel distance in parking system routing. They aim to minimize distance traveled by a driver looking to find a free parking spot, using the algorithm, an optimized route is determined for the parking spot. In [131,132,133] the ant colony optimization algorithm is used to determine the best route in a generic traffic scenario where cars can communicate with road side units in a Vehicular Adhoc NETwork (VANET) architecture. Routing for public transport is performed by [134,138] in a connected vehicle scenario aiming to minimize travel time. An economic objective approach to traffic routing is taken by the authors of [127,136,137] who minimize the total cost of the trip. Mao [127] also include traffic congestion and travel time as well in their computation. Hassoune et al. [139] work on a parking guidance using the ant colony optimization algorithm to reduce traffic congestion and minimize distance. Constraints for traffic routing are related to the road network allowing travel in specific directions, signaling and travel time. Within smart transportation, IoT nodes are used to determine occupied parking spaces and this data is used for routing applications in parking. Traffic routing is based on vehicle to vehicle and vehicle to infrastructure communication provided by VANETs within the IoT framework. These systems enable cars to exchange data with each other and also with fixed infrastructure on the roads. This discussion is also presented in Table 15 and the data setups for the covered work are presented in Table 16.

## 4. Recommendations

This survey discussed the application scenario for optimization algorithms within the IoT based Smart Cities in terms of objectives, constraints and formulations. There are several takeaways from this exercise. The first aspect observed was the lack of standardized datasets being utilized by the methodologies covered as discussed in the various sections. This limits the ability to effectively compare proposed methodologies for a similar problem. This issue was less observed for the case of Smart Grids where standardized network architectures were used. The use of standardized test sets would enable a fair comparison of competing methodologies. Another aspect would be the use of more detailed statistical analysis of experiment data such as running time than mean, standard deviation etc as has been mentioned by [7]. Such analysis would help to understand better the effects of different constraints on the algorithm better also help with comparative analysis with other methodologies. For the nature of coverage herein, it would contribute to possibly looking at performance of cross-smart city component applications which are similar.

## 5. Future Work

While this review presents a coverage of the current state of IoT enabled Smart City optimziation applications, there are several avenues of future work that have been identified as well.

### 5.1. Novel Applications

While combinatorial optimization algorithms have found wide ranging applications in all aspects of a city’s operations for e.g., in planning [140,141] and scheduling [142], it is expected that as more aspects of a city are instrumented and data gathering takes place, applications of optimization algorithms which work on real-time measurements will be further developed. With newly instrumented systems, one could also leverage machine and deep learning algorithms for predicting a variable of interest and then utilize optimization algorithms for a given application. Such a combination could spearhead optimization application development. Apart from prediction, machine learning could be used for classification purposes too in conjunction with combinatorial optimization schemes. One such application could be in the industry where worker activity recognition [143] is performed and such data is collected for then scheduling operations in cooperation with automated machine processes using heuristics.

### 5.2. Hybrid Algorithms

The aim of hybrid methodologies is to combine the best performance characteristics of different algorithms to reach to an optimal solution for an optimization problem. There have been several works which combine multiple optimization techniques. For e.g., the authors in [144] use a combination of a PSO and GA to solve the ambulance location and allocation problem. They do this in a subproblem form with the objective being the minimization of the mean waiting time of the injured people and the response time between stations and affected areas. The constraints are balance of flow, cost of open stations, number of ambulances given to an areas satisfying its requirements, one affected area served by one station, station ambulance capacity is respected and that no ambulances allocated if station is closed. It is expected that work towards hybrid algorithms algorithms will increase the applicability of combinatorial optimization in smart cities. Such a hybrid system has been used in Smart Agriculture by [145] also utilizing a GA and a modified PSO algorithm.

### 5.3. Novel Nature Inspired/Heuristic Algorithms

Ant colony, Genetic algorithms and Particle Swarm Optimization, Differential Evolution and Artificial Bee Colony algorithms for solving Smart City optimization problems were considered during the survey. However, there were some attempts that were based on other evolutionary or collective behavior of other living organisms. Examples of such algorithms include the use of shuffled frog leaping optimization, graywolf optimization [146] for power management and also for traffic routing [147], earthworm optimization [148] for power management in smart grid, vehicle routing using simulated annealing [149] and several different ones for home energy management [150] and elephant herding optimization [151] as well in addition to others.

### 5.4. Distributed Optimization Scheme

As the computation power at the edge increases, the methodologies which utilize a distributed optimization scheme to fully utilize the IoT capabilities they operate in can potentially provide better performance. Herein, there could be multiple objectives and each can be optimized at a lower level before optimization is performed at a higher one are bound to increase. One such example was suggested by Tang et al. [122] who carry out distributed optimization in a fog and cloud architecture. First, fog nodes optimize phase timings within a single cycle and if the vehicles exceeding number increases a threshold, the results are sent to the central controller to further optimize over different cycles so that a traffic jam is avoided or alleviated.

### 5.5. Use of Reinforcement Learning

Reinforcement learning (RL) has the potential to provide solutions to combinatorial optimization problems as covered in [152]. The idea is to use machine learning and reinforcement learning to get rid of human created heurists which may lead to optimizations towards local optimums. Agents can be trained to search for these heuristics to automate the process. ML and RL methods have been observed to be faster compared to metaheuristic methods for solving optimization problems as noted by [152], especially for large problems. Such methods could be useful for applications within the IoT based Smart City landscape. It must be noted however that the usage of RL and ML towards combinatorial optimization problems is still a growing research area. An example of such use is its use for traffic signal control as described in [153].

## 6. Conclusions

**Figure 9:**
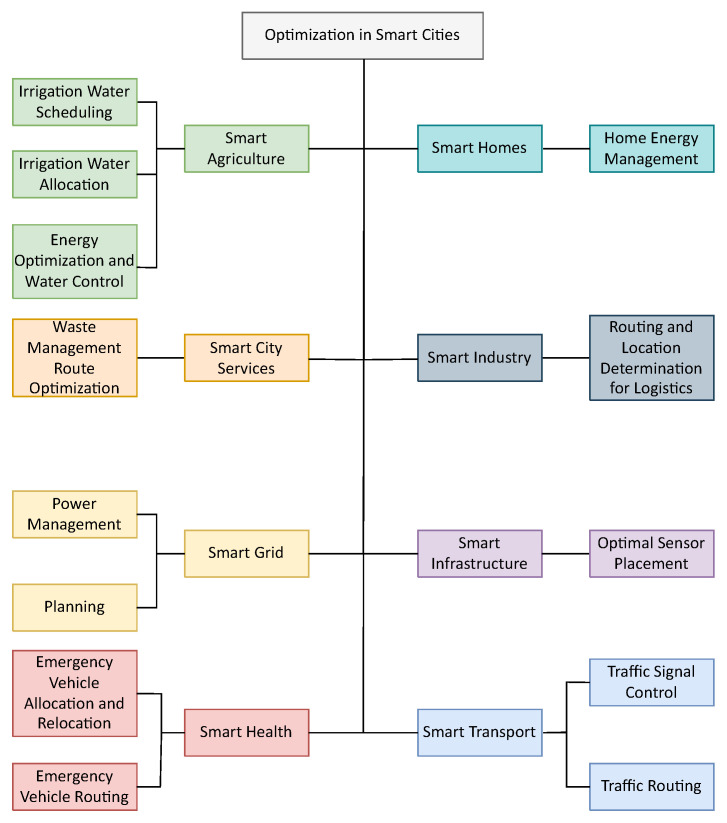
Optimization applications in IoT based Smart Cities.

This paper provides coverage of the application of five popular computational algorithms in the IoT enabled Smart City. It provides a mapping of the various applications to the specific smart city domain as well as highlights the different formulations of the objective function used to solve the considered problem. This coverage is provided in terms of the number of objectives as well as whether the problem was solved as a single objective, in a hierarchical manner or otherwise. It also highlights the constraints used by the researchers in solving the problem which is an important aspect as constraints are governed by the application at hand. An overview of the mapping of various smart city optimization applications derived from this work is provided in Figure 9.

**Figure 10:**
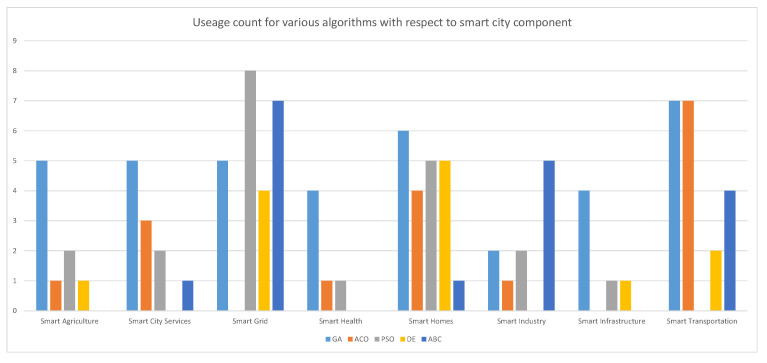
Count of different algorithms used with respect to Smart City Component.

Figure 10 illustrate the distribution counts for each of the algorithms considered in this survey. It was found that genetic algorithms was found to be most commonly used optimization scheme as can be seen from Figure 10 and was used a total of 33 times in the approaches covered in the survey as has been observed by. This inspite of the fact that GAs are more computationally intensive than PSO with the latter being faster as well [154]. However, it must be noted that the performance of any optimization algorithm is problem dependent [154]. The PSO algorithm was used the most for smart grid applications whereas ACO and GA were equally used for Smart Transportation. When looking at the difference between the uses of bio-inspired (PSO, ACO, ABC) and nature-inspired (GA, DE) algorithms, it was observed that bio-inspired algorithms were used more times at 53 vs. 45 respectively as the proposed technique. This indicates that nature inspired algorithms, even though relatively newer, are getting increasing traction for use in various applications relating to Smart Cities.

**Figure 11:**
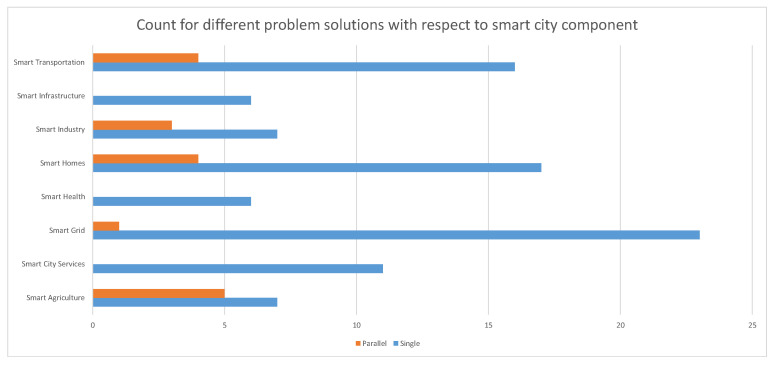
Solution scheme for problems with respect to Smart City Component.

Figure 11 illustrates the counts for the different ways in which the objective function was solved. It can be observed that even though there were nearly similar number of multiple and single objective function formulations (59 and 51 respectively), the solutions for these were mostly derived as a single problem. This meaning that the objectives were combined in some form (such as weighted combination of two or more objectives).

Another interesting observation from this review was on the formulations of similar standard combinatorial optimization problems within different smart city domains. For e.g., the vehicle routing problem exists in smart health (emergency vehicle routing), smart transportation (traffic routing and public transport routing) as well as in smart industry (routing for logistics). While, the objective of the routing problem in various papers was observed to target time incurred for the trip, the constraints incorporated domain knowledge in to the problem. That is, routing in the smart industry included constraints on visiting all depots while for smart health and smart transport, constraints included speed and road traffic. These insights highlight the difference in working on similar optimization problems in different smart city domains.

This review will help researchers in the field of computational optimization for smart cities to develop better problem formulations for the problems encountered in IoT based smart cities. It will also provide new researchers starting in the field by presenting them with an overview of the optimization scope within the IoT supported Smart City domain.

## Figures and Tables

**Table 1:** Optimization in Smart Agriculture.

**Application**	**Algorithm**	**Single/Parallel Problems**	**Objectives**	**Constraints**
Irrigation Management (Irrigation Water Scheduling)	ACO [13]	Single	Maximizing net return on crop	Constraint on water availability
	PSO [14]			Capacity of irrigation system
				Water savings should be more than deficiency
	GA [17]	Single	Minimize water fluctuations and difference between the time of water demand and need	Finite canal capacity
				Maximum rotation time limitation
	GA [15]	Parallel	Maximize yield, global and local water use efficiencies	Constraint on irrigation interval
				Minimum and max irrigation amount
	GA [16]	Parallel	Minimize leakage loss both individually and overall	Flow capacity limited by maximum
				Irrigation time constraint
				Net discharge constraint
				Total flow rate should be sum of individual flowrates
Irrigation Management (Irrigation Water Allocation)	DE [18]	Single	Minimize irrigation water allocated and maximizes net benefits	Constraint on the land area available
				Minimum and max planting areas for crops
				Limited water available for the farm
	PSO [19]	Parallel	Minimize deviation in the different channels, water seepage in the distribution channels	Time
				Water quantity constraints
	GA [22]	Parallel	Maximize benefit to regional water supply, minimize water deficit groundwater exploitation in regions	Water supply quantity constraints for annual water, ground water
Irrigation Management (Energy Optimization)	GA [20]	Parallel	Minimize energy cost while maintaining water supply for plants	Limited energy available
				Water volume maintained in storage tank, fish pond
	GA [21]	Single	Minimize sum of squared water shortage	Annual water availability in reservoir
				Water rights of replenishment pumping station
				Water rights of the irrigation pumping station
				Operational rule constraints
Irrigation Management (Water Control)	GA [25]	Single	Maximize yield	Minimum and maximum water depth limits
				Min and max soil moisture
Irrigation and Fertilizer Management	GA [24]	Single	Maximize economic profits and environmental benefits	Limits on the demand of water for each crop
				Total water does not exceed available
				Total fertilizer doesn’t exceed availability
				Water allocation should be positive

**Table 2:** Data setup used for Smart Agriculture Optimization.

**Data Type**	**Papers**
Self-collected/Presented	[14,16,17,20,24,25]
Government and private agencies	[13,14,15,16,17,18,19,25]

**Table 3:** Optimization in Smart City Services.

**Application**	**Algorithm**	**Single/Parallel Problems**	**Objectives**	**Constraints**
Waste Management Route Optimization	ACO [27]	Single	Minimization of distance	Road Network is fixed
	GA [28,29,31]			Each dumpster served by one truck only
				Trucks leave depot to go to landfill
	PSO [30]			Routes are continuous
	ABC [32]	Single	Minimize CO${}_{2}$ emissions	Capacity constraint for bins as well as trucks
	ACO [33]	Single	Minimize total travel time	Trucks leave a depot empty
	GA [34,35]			Bins needs to be fully emptied by trucks
				Vehicle start depot and end at landfill
	PSO [36]			Demand should not exceed capacity
	ACO [37]	Single	Minimize travel cost and total usage cost of vehicles	Subtour elimination
				Jobs should finish within a given deadline

**Table 4:** Data setup used for Smart City Services Optimization.

**Data Type**	**Papers**
Self-collected/Presented/Generated	[27,28,29,31,33,35,37]
Government Agency	[34,36]
Dataset	Capacitated VRP datasets [38] by [30], Capacitated VRP Instances [39] by [32]

**Table 5:** Optimization in Smart Grid.

**Application**	**Algorithm**	**Single/Parallel Problems**	**Objectives**	**Constraints**
Power Management (Improve Grid Performance)	ABC [40]	Single	Minimize active power loss, volage deviation and voltage stability index (L-index)	Power flow constraints
	GA [42]			Restriction on power source installations and other components related to power structure
	PSO [44,46]	Single	Minimize power loss	Generation and other component operations within limits
	GA [43]	Single	Minimize average percentage of loadability of the lines, active power loss, reactance of transmission line	Limitation on values of bus voltage
				Transmission line capacity, generator active and reactive power.
	ABC [41]	Single	Minimize cost for maintaining thermal and voltage stability and lower asset management of distribution networks	Active and reactive power must be balanced
				Limits on voltage and load maximum
				ESS max charging and discharging constraints
	PSO [45]	Parallel	Maximize annual profit by reducing charges for annual energy losses, peak power loses etc	Constraint on the node voltage (soft)
			Minimize power loss for the network reconfiguration	Power injected by DER and SG within limit
				Power generated at a given node has a limit
				For reconfiguration:
				Radial topology,
				Node voltages has a max hard constraint
Power Management (Distributed Energy Resource Management)	ABC [50,51,52]	Single	Minimize total cost	Power generation by renewables within limits
	DE [53,54,55]			Battery charge and discharge limits and system reliability
	GA [47,48]			Power balance constraint (generated equal to consumed)
	PSO [58,59,60]			Specific loads are interruptible
				Constraints on the efficiencies of the sources
	DE [56]	Single	Minimize cost and emission	
	ABC [63]	Single	Minimize cost and power imported from outside micro-grid	Power flow constraints for the DER
	GA [49]	Single	Minimization of cost of energy and life cycle emissions (CO${}_{2}$ and energy stored in batteries or converted by renewable sources during process of satisfying load requirements)	Constraints on battery capacity
				System reliability constraint
				Energy produced equal or greater sthan required
	PSO [57]	Single	Minimize reliability cost, cost of electricity production and operation environmental impact ()using renewable factor)	
Expansion of distribution network	ABC [61]	Single	Minimize cost of network expansion, active losses and loss of load and generation	Power flow and active power balanced
				Power generation limits
				Number of transmission line limits
	PSO [62]	Single	Minimize number of PMUs	SG Network should be observable

**Table 6:** Data setup used for Smart Grid.

**Data Type**	**Papers**
Self-collected/Presented/Generated	25 Bus networks [49,53,55,56,57,58,59,60,63]
Government Agency/other research work	[48,50,51,52,57,61]
Dataset/Standard Network	IEEE 14 Bus [42,62] IEEE 30 Bus [40,42,43] IEEE 33 Bus [41,44,45,54] IEEE 37 Bus [47] IEEE 57 Bus [40,42,62] IEEE 69 Bus [45] 119 Node system of [46,64]

**Table 7:** Optimization in Smart Health.

**Application**	**Algorithm**	**Single/Parallel Problems**	**Objectives**	**Constraints**
Emergency Vehicle Allocation and Relocation	ACO [71]	Single	Minimize lateness	Ambulance from nearest hospital is dispatched
	GA [72]			Speed of ambulance
				Total number of ambulance limits
	GA [73]	Single	Minimize average waiting time of ambulances	Balance constraints on exit and entry volumes
				Flow conservation constraints
	GA [74]	Single	Minimize total cost in money and time	
Emergency Vehicle Routing	PSO [76]	Single	Minimize travel time, road length traveled, density of vehicles on the road	Road connections are specific
	GA [75]	Single	Minimize the entrance time of emergency vehicle by changing the order of vehicles going through intersections	Constraint on the difference between arrival times of current and previous vehicles and on the entrance time of the vehicle

**Table 8:** Data setup used for Smart Health.

**Data Type**	**Papers**
Self-collected/Presented/Generated	[71,72,73,74,75,76]
Government Agency/other research work	[72,73,74]

**Table 9:** Optimization in Smart Homes.

**Application**	**Algorithm**	**Single/Parallel Problems**	**Objectives**	**Constraints**
Home Energy Management	ACO [77]	Single	Minimize cost and waiting time	Comfort needs to be maintained
	ACO [80]	Parallel	Minimize cost and peak to average ratio	Power flow constraints
	ACO [81]	Single	Minimize cost and peak to average ratio	Maximum energy capacity constraint
	DE [82]			Device counted that can be shifted is positive
	PSO [83]			Number of devices shifted at any time should not be more than the available number of controllable devices
	GA [95]	Single	Minimize peak to average ratio for load shaping	Load shaping, redistribution of load in a flexible manner
	GA [84]	Single	Minimize ratio of operating cost and load factor	Charging and discharging of batteries
				Complete load transfer and load clipping limits
	DE [85]	Single	Minimize electricity cost, peak to average ratio of power and discomfort minimization of users	Constraints on PV supply limits
	ACO [86]			State of charge and rate of discharge of battery
	DE [87]	Single	Minimize electricity cost and discomfort	Time of operation within specified limits
	PSO [89]			Temperature, air quality, illumination and energy should be within maximum limits
	GA [63,88,96]	Parallel		A given appliance must be on for specified times of the day
				Power limits to be followed
	ABC [78]	Single	Minimize cost of electricity	Appliances for comfort have fixed times
	DE [90,92]			Some appliances cannot be delayed
	GA [93]			Power balance constraints
	PSO [79,94]			Surplus solar power sold back to distribution system
				Maintain zero net energy in building
				Time constraints
				Load safety factor
				Load phases of appliances fulfill energy requirements
				Comfort needs to be maintained
				Peak to average power ratio balancing
	PSO [91]	Single	Minimize energy bill and cost associated with KWH curtailment	Power values within limits, battery charge and discharge limits

**Table 10:** Data setup used for Smart Homes.

**Data Type**	**Papers**
Self-collected/Presented/Generated	[63,77,78,79,80,81,82,83,84,85,87,88,92,93,94,96]
Government Agency/other research work	[79,80,81,85,89,90,91,95,96]

**Table 11:** Optimization in Smart Industry.

**Application**	**Algorithm**	**Single/Parallel Problems**	**Objectives**	**Constraints**
Location determination for sites	ABC [100]	Single	Minimize transportation and hub establishment cost	Single allocation for each demand node
				A given number of hubs are established
				Covering radius constraint
				Time reliability constraint
	GA [101]	Parallel	Minimize distribution cost and maximize profit	Load capacity meets needs of customers
				A delivery vehicle can only be delivered when it receives a task
				Capacity constraints
Routing for Logistics	ABC [106]	Parallel	Minimize distance travelled, CO${}_{2}$ emissions, number of vehicles used	Every customer visited only once
				Every vehicle visiting a location must leave it too
				Ensure route continuity
				Demands of any route must not exceed capacity
				Edges satisfying time window constraint are allowed.
	ABC [107]	Single	Minimize total transportation distance	Each customer served only once
	GA [105]			Route should start and end at the same depot
				Served demand of each vehicle does not exceed capacity limit
	ACO [102]	Single	Minimizing total cost	Each customer served only once
	PSO [103]			Dispatched vehicles not more than available
	ABC [104]			Vehicle routes don’t contain disconnected routes
				Customer demand shouldn’t be larger than vehicle capacity
	ABC [109]	Single	Minimize travelling time	Vehicle load constraint
				Subtours not allowed
				Speed, time and distance
				Maximum number of vehicles on a route
				Each customer served by one vehicle
				Vehicle number max limit
	PSO [108]	Parallel	Minimize fuel consumption and travel time	Each customer serviced by only one vehicle
				Continuity in route
				Vehicle load conservation between nodes,
				First in first out proper when traveling time is computed
				Time taken for customers as stated,
				Maximum time for servicing
				Vehicle capacity constraint
				Depot is the first and final destination of each vehicle

**Table 12:** Data setup for Smart Industry.

**Data Type**	**Papers**
Self-collected/Presented/Generated	[100,101,105,109]
Government Agency/other research work	[102,104,106,107,109]
Dataset/Standard Network	Test instances in [110] used by [103,108]

**Table 13:** Optimization in Smart Infrastructure.

**Application**	**Algorithm**	**Single/Parallel Problems**	**Objectives**	**Constraints**
Sensor placement	GA [111]	Single	Minimize measurement error and measurement cost	
	PSO [112]	Single	Maximize reconstruction accuracy and robustness of transfer relationship between deformation displacement and surface strain (formulated as a minimization problem for negated accuracy and robustness)	Sensor placements within predefined range and angles
	GA [113]	Single	Minimize the ratio of sensor placement performance to redundancy information	Sensor placement is permitted on chosen location
	GA [114]	Single	Minimize the MAE between the system and the estimated response (global error) and minimize the maximum difference between the system and its estimated response (local error)	Sensor locations are from a set of predefined locations
	DE [115]	Single	Maximize quality of coverage, lifetime, connectivity uniformity of sensor nodes and cluster heads and reliability	Constraint on the number of cluster heads associated with each sensor node and cluster head
	GA [116]	Single	Minimize cross correlation of the sensing network	Sensor placement is permitted on chosen location

**Table 14:** Data types for Smart Infrastructure.

**Data Type**	**Papers**
Self-collected/Presented/Generated	[111,112,113,114,116,117,118,118]

**Table 15:** Optimization in Smart Transportation.

**Application**	**Algorithm**	**Single/Parallel Problems**	**Objectives**	**Constraints**
Traffic signal control	ABC [138]	Single	Minimize travel time	Interval of feasible green time length values
	ABC [128]			Interval of feasible offset time length values
				Constraints on cycle lengths
	ABC [119,120]	Single	Minimize time delay	Only one active stage
	GA [121,122]			Flow dynamic constraint
	GA [123]	Parallel	Minimize time delay and also achieve traffic network equilibrium	Link volume constraint
				Constraints on duration of green/red phases
				Offset phase duration
				Minimize average travel time.
				Relationship between route and link flows need to be maintained as defined
	GA [124]	Single	Minimize vehicle emissions and travel time for vehicles	Sum of green time of each phase is equal to total available green time
				Green time is set by a lower bound
	GA [126]	Parallel	Minimize delay, and exhaust emission and maximize traffic capacity (formulated as minimization problem)	Cycle length of signals has minimum and maximum limits
Traffic Routing (Parking System)	ACO [129]	Parallel	Minimize distance with bend straightening and turn reduction	Bend straightening and turn reduction
	ACO [139]	Parallel	Reduce traffic flow and shortest distance towards parking	
	GA [130]	Single	Minimize distance	Specific prefixed routes possible for free parking
Traffic Routing (Road Traffic)	ACO [131,132]	Single	Minimize distance, minimize congestion	Follow roadmap
	ACO [133]	Single	Maximize flow	
	ACO [134]	Single	Minimize travel time	Constraint on relationship between green time lengths cycle length, offset on the network calculation
	GA [135]			Interval of feasible green time length values
				Interval of feasible offset time length values
				Specific road segments
				Connected constraints on the values of time taken for vehicles
	DE [136]	Single	Minimize travelling cost and rental cost	Each bus has one employee
				Employees can be assigned when stop is available
				Bus stop assigned when bus is in use
				Constraint on distance of bus stop from employee home and more
	DE [137]	Single	Minimize total cost	Road network connections followed
				Solutions contains correct number of routes
	ACO [127]	Single	Minimize transit time, travel distance, road congestion and traffic expenses	Variable value constraints

**Table 16:** Data types for Smart Transportation.

**Data Type**	**Papers**
Self-collected/Presented/Generated	[119,120,121,122,123,124,126,128,129,130,131,132,133,135,138,139]
GovernmentAgency/other research work	[119,121,123,124,128,130,134,135,136,137]

## Data Availability

Not applicable.
